# Engineering A-kinase Anchoring Protein (AKAP)-selective Regulatory Subunits of Protein Kinase A (PKA) through Structure-based Phage Selection*[Fn FN2]

**DOI:** 10.1074/jbc.M112.447326

**Published:** 2013-04-26

**Authors:** Matthew G. Gold, Douglas M. Fowler, Christopher K. Means, Catherine T. Pawson, Jason J. Stephany, Lorene K. Langeberg, Stanley Fields, John D. Scott

**Affiliations:** From the ‡ Howard Hughes Medical Institute and; the Departments of § Pharmacology,; ¶ Genome Sciences, and; ‖ Medicine, University of Washington School of Medicine, Seattle, Washington 98195

**Keywords:** AKAP, Cell Biology, Peptide Arrays, Phage Display, Protein Kinase A (PKA), Compartmentalization, Structure-based Design

## Abstract

PKA is retained within distinct subcellular environments by the association of its regulatory type II (RII) subunits with A-kinase anchoring proteins (AKAPs). Conventional reagents that universally disrupt PKA anchoring are patterned after a conserved AKAP motif. We introduce a phage selection procedure that exploits high-resolution structural information to engineer RII mutants that are selective for a particular AKAP. Selective RII (R_Select_) sequences were obtained for eight AKAPs following competitive selection screening. Biochemical and cell-based experiments validated the efficacy of R_Select_ proteins for AKAP2 and AKAP18. These engineered proteins represent a new class of reagents that can be used to dissect the contributions of different AKAP-targeted pools of PKA. Molecular modeling and high-throughput sequencing analyses revealed the molecular basis of AKAP-selective interactions and shed new light on native RII-AKAP interactions. We propose that this structure-directed evolution strategy might be generally applicable for the investigation of other protein interaction surfaces.

## Teaser

**Background:** PKA is confined to subcellular compartments by A-kinase anchoring proteins (AKAPs).

**Results:** A structure-based phage-directed evolution strategy has yielded modified PKA regulatory type II subunits with AKAP-selective binding properties.

**Conclusion:** Engineered R_Select_ proteins preferentially target particular AKAP-PKA interfaces

**Significance:** R_Select_ subunits are tools to distinguish and manipulate subpopulations of anchored PKA.

## Introduction

Cellular regulation often requires that broad-spectrum enzymes are organized in a manner to optimally transduce chemical and ionic signals. Anchoring and scaffolding proteins promote such signaling efficacy by placing enzymes near substrates and by insulating signaling units from one another (1). Most mammalian cells express 10–15 different A-kinase anchoring proteins (AKAPs)^3^ that restrict the location of PKA to particular intracellular membranes or organelles (2). Similarly, phosphatase-targeting subunits compartmentalize protein phosphatases to spatially restrict signal termination (3). A hallmark of both classes of signal-organizing proteins is the presence of short docking sequences that associate with binding pockets on their partner enzymes (4). Structural biology and peptide array analyses have identified a consensus motif ( L*xxx*A*xx*IV*xx*AI*xx*A) that forms the aliphatic face of an amphipathic helix on each AKAP (5, 6). This region inserts into a hydrophobic groove on the surface of the docking and dimerization (D/D) domain of the PKA regulatory type II (RII) subunit dimer (5, 6). This provides a molecular mechanism to sequester anchored pools of PKA in proximity to upstream activators (7) and in the vicinity of downstream substrates (8). As a result, this broad-spectrum kinase can simultaneously and independently operate at different locations within the same cell. Likewise, the catalytic subunit of protein phosphatase 1 associates with proteins bearing the motif RV*x*F (9, 10), whereas protein phosphatase 2B (calcineurin) docks to proteins bearing the PI*X*IT motif (11, 12).

In recent years, reagents have been developed for the manipulation of localized signaling processes inside cells. These include a version of the yeast Ste5 scaffolding protein with an artificial binding site that reshapes the relay of information through three-tier MAPK cascades (13) and a genetically encoded photoactivatable Rac GTPase that permits the inducible control of actin-based cell movement (14). Perhaps the most established form of targeted enzyme modulation has exploited the RII-AKAP interface (5, 6). The original PKA-anchoring disruptor peptide Ht31 (15), derived from the anchoring helix of AKAP-Lbc, was used to demonstrate a role for PKA anchoring in a range of biological processes, including phosphorylation-dependent modulation of synaptic transmission (15), hormone-mediated insulin secretion from β-islets (16), and maintenance of ocular lens transparency (17). A second generation of anchoring disruptor peptides was derived from spot array screens of typically 400 peptide variants of the RII-binding sequence (5). Reagents developed by this approach are more potent than Ht31 (18) and distinguish between anchored type I (19) and type II (5) PKA R subunits. However, the overriding limitation of these generic PKA-anchoring disruptors is their inability to discriminate between the contributions of individual AKAPs in the control of anchored signaling events. In this study, we introduce a structure-based phage selection strategy that engineers RII subunit fragments that bind selectively to individual AKAPs. These genetically encoded AKAP-selective probes provide tools to manipulate PKA-responsive processes that are directed by individual anchoring proteins. The approach is applicable to other shared protein interaction surfaces where high-resolution structural information is available.

## EXPERIMENTAL PROCEDURES

### 

#### 

##### Protein Purification

Purification of ∼60-amino acid regions containing RII-anchoring helices was successfully attempted for 10 of 16 different AKAPs. The 16 AKAPs comprise AKAP1–14 as designated by the HUGO Gene Nomenclature Committee, MAP2, and WAVE1. The fragments were synthetically amplified (20) and ligated into pGEX6P1 for bacterial expression as fusions at the C terminus of GST (supplemental Table S1). GST-AKAP fragments were purified by affinity on glutathione-Sepharose and by gel filtration. R_Select_AKAP18FL (where FL is full-length) was expressed and purified using a similar procedure, but with elution from glutathione-Sepharose by incubation with PreScission protease (GE Healthcare) rather than l-glutathione. R_Select_ D/D domains (residues 1–45) were expressed with an N-terminal PreScission-cleavable His_6_ tag and C-terminal V5 tag in pET-28m and purified by nickel affinity and gel filtration chromatography. Overlays were performed with 1:10,000 (w/v) RII/R_Select_ subunits in TBS/Tween 20 supplemented with 10% milk. Binding was detected with 1:5000 (w/v) HRP-conjugated anti-V5 antibody (Invitrogen).

##### RII Mutant Phage Library Generation

RII phage were generated using the T7Select® system (Novagen). PKA RIIα (residues 1–45) with the 10-amino acid N-terminal linker SGSGSSGGSG was inserted after Asn-351 of the T7 capsid protein by ligation into T7Select10-3b EcoRI/HindIII vector arms (Novagen). Primers were used to amplify the following DNA template prior to ligation: GTTCTTCTGGTGGTTCTGGTATGTCTCAC**ATC**CAG**ATC**CCGCCGGGTCTG**ACC**GAACTGCTG**CAG**GGTTACACCGTTGAAGTTCTGCGTCAGCAGCCGCCGG. The boldface underlined bases code for RII positions 3, 5, 10, and 14. The template oligonucleotide was synthesized with a random mixture (25% of each nucleotide) at these 12 positions (TriLink BioTechnologies) to generate the RII variant library. Because there are 4^12^ total DNA variants, the library coverage = 1 − (16777215/16777216)*^n^*, where *n* is the packaging number. 8.8 × 10^7^ inserts were packaged into the phage, so ∼99.5% of all possible DNA variants are represented in the library. Given the redundancy of the genetic code, the coverage of total protein variants is approaching 100%.

##### Phage Selection

All steps were performed in phage wash buffer (25 mm Tris (pH 7.2), 150 mm NaCl, 0.05% Tween 20, 2 mm DTT, and 0.5 mm EDTA). Prior to each round of selection, 0.125 μl of glutathione magnetic beads (Pierce) was incubated with 0.1 μg of a single GST-AKAP fusion protein. After washing with phage wash buffer (3× 1 ml), ∼10^10^ RII phage were input in each round of selection in the presence of 40-μg peptide aliquots corresponding to the anchoring helices of the 15 AKAPs that were not immobilized (supplemental Table S1). Although AKAP identification is ongoing, we reasoned that this comprehensive list of competitors would drive the selection toward the most selective RII variant for each AKAP. Following washing with phage wash buffer (6× 1 ml), phage were eluted by a 2-h incubation with 0.5 μg of PreScission protease in 100 μl of phage wash buffer. Eluates were incubated for 10 min with 2 μl of glutathione magnetic beads to remove residual PreScission protease and then amplified to serve as the input in the next round of selection. The RII mutant sequences of phage present in eight plaques from the sixth/eighth round of selection were amplified by PCR and subjected to Sanger sequencing (supplemental Table S2). For screens with medium-selection pressure, the quantity of each free competitor AKAP peptide was reduced to 2 μg. When screening in the absence of competitors, 1 μg of GST-AKAP bound to 0.5 μl of glutathione magnetic beads was used as the bait.

##### Amplified Luminescent Proximity Homogenous Assay (AlphaScreen)

Assays were performed in a 40-μl total volume with buffer consisting of 20 mm HEPES (pH 7.2), 100 mm NaCl, and 0.1% BSA. Dissociation constants (*K_d_*) for AKAP-PKA D/D domain interactions were determined using the AlphaScreen competition binding assay. Biotinylated R subunit D/D domains (0.3 nm, “tracer”) were incubated with fusions of GST and the RII-binding regions of AKAP2 or AKAP18 (3 nm, “target”) with increasing concentrations of the untagged R subunit D/D domain. At these concentrations, the IC_50_ for disruption with the untagged R subunit D/D domain approximates the *K_d_* of its interaction with the AKAP target. To quantify R_Select_AKAP18 and R_Select_AKAP2 selectivity, fusions of GST and the RII-binding regions of either AKAP2 (1 nm) or AKAP18 (1 nm) were incubated with biotinylated R_Select_ subunit D/D domains (10 nm) in the presence of increasing concentrations of competitor AKAP peptides. For GST-AKAP2, the competitor peptides consisted of an equimolar mixture of AKAP18, AKAP-Lbc, and AKAP150. For GST-AKAP18, the competitor peptides consisted of an equimolar mixture of AKAP2, AKAP-Lbc, and AKAP150. In all cases, following a 1-h incubation at 4 °C, streptavidin donor beads (20 ng/μl) and anti-GST acceptor beads (20 ng/μl) (PerkinElmer Life Sciences) were added. Assays were incubated for 2 h prior to measurement of fluorescence at 570 nm. Analysis was performed by fitting data according to one-site competition in GraphPad Prism.

##### Cell Culture and Pulldown, Imaging, and FRET Experiments

HEK293 cells were cultured in DMEM supplemented with 10% (v/v) fetal bovine serum and penicillin/streptomycin. Cells were transfected overnight using TransIT-LT1 (Mirus Bio LLC). Pulldown experiments were performed with 2 μg of anti-FLAG or anti-V5 antibody (Sigma) supplemented with 0.4 μg of R_Select_AKAP18FL in the presence or absence of 2.5 μg of Ht31 peptide (10 μm) as appropriate. Associated PKA C subunits were eluted by incubation with 1 mm cAMP. Staining of FLAG-AKAP18 and AKAP2-V5 was performed by incubation with rabbit anti-V5 and mouse anti-FLAG primary antibodies and Alexa Fluor 568-coupled donkey anti-rabbit and Alexa Fluor 488-coupled goat anti-mouse secondary antibodies (Invitrogen). Imaging was performed with a Zeiss LSM 510 META confocal microscope. For FRET measurements, dual-emission FRET images were obtained using a Leica DMI6000 B microscope equipped with a dual-view image splitter (Photometrics), S470/30 and S535/30 emission filters, and a 505DCXR dichroic mirror (Chroma). MetaMorph imaging software was used to quantify the FRET images using the fully specified bleed-through correction method.

##### Structural Modeling

Models of R_Select_AKAP complexes were generated using the RII D/D-AKAP-*is* structure (5) as a template. The mutant protein complex structures were manually refined using the 2*F_o_* − *F_c_* electron density map of the RII D/D-AKAP-*is* crystal structure as a reference in Coot (21). Structural representations were generated using PyMOL (Schrödinger).

##### Illumina Library Preparation, Sequencing, and Quality Filtration

Phage library DNA was isolated by phenol/chloroform extraction and ethanol precipitation. 50 ng of phage DNA was amplified using the DF-97_PCR_long_p1F and DF-154_PCR_p1R primers (supplemental Table S3). Libraries prepared from the fourth round of selection of AKAP18 with competitors, the third round of selection without competitors, and the input phage were sequenced using a HiSeq 2000 system (Illumina) with the DF-154_SEQ_F and DF-154_SEQ_R primers (supplemental Table S3).

##### High-throughput Sequencing Data Analysis

Sequencing data analysis was carried out using the Enrich software package (22, 23). Standard Enrich output was generated using the configuration parameters detailed under supplemental “Methods.” An average Illumina quality score was calculated for each read in a given set of paired-end reads, and pairs that had an average Phred quality score of <20 for either read were removed. Read pairs were merged into a single sequence, and sequences failing to meet several quality criteria were discarded. Unique sequences (variants) from each library were identified, and their frequency was computed. Variants supported by less than 10 reads were removed. Log-transformed variant enrichment ratios (*E*) were calculated as described (37). Unlinked mutation scores for the *i*th position and the *j*th amino acid (*U_i_*_,_*_j_*) were calculated as follows (Equation 1).

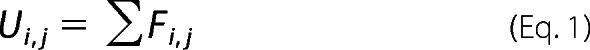
 These were divided by the sum of all unlinked mutation scores to the produce unlinked amino acid frequency for the *i*th position and the *j*th amino acid (*Fu_i_*_,_*_j_*) (Equation 2).

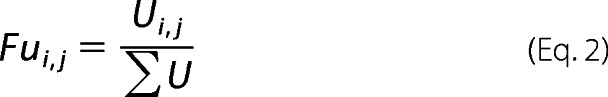
 The unlinked enrichment ratio for the *i*th position and the *j*th amino acid between the input library and the *X*th round (*E*_unlinked,_*_i_*_,_*_j_*_,_*_X_*) was calculated as follows (Equation 3).

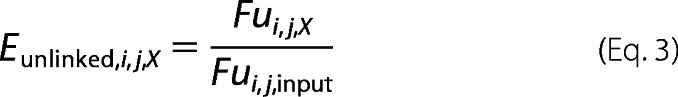
 The selectivity index was calculated for the *i*th position and the *j*th amino acid between the AKAP18 library in the presence or absence of competitor AKAP peptide (*SI_i_*_,_*_jj_*) as follows (Equation 4).

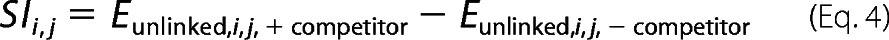
 Extended experimental procedures are provided under supplemental “Methods.”

## RESULTS

### 

#### 

##### Rational Structure-based Construction of an RII Variant Phage Library

**FIGURE 1.**
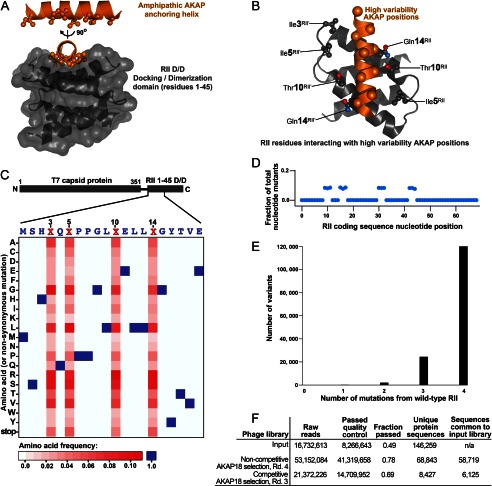
**Construction of RII variant phage library.** *A*, structural representation of low-variability aliphatic residues on one face of the AKAP anchoring helix (*orange*) and the reciprocal binding surface in the RII D/D domain (residues 1–45; *gray*). *B*, residues in PKA RII that contact AKAP positions of higher variability (*orange spheres*). *C*, frequencies of each amino acid at every position in the first 20 amino acids of RII expressed on the surface of the phage in the input library. *D*, distribution of mutant nucleotides within the RII coding sequences of the input RII phage library. Mutations in wild-type RII coding sequence were restricted to codons 3, 5, 10, and 14. *E*, distribution of single, double, triple, and quadruple RII mutants in the input phage library. *F*, high-throughput sequencing statistics of RII variant phage libraries. *Rd.*, round; *n/a*, not applicable.

AKAPs contain a 16-residue sequence that is necessary and sufficient for PKA anchoring (18, 24). Conserved aliphatic side chains within this sequence provide the hydrophobic face of an amphipathic helix. These residues participate in van der Waals interactions with a reciprocal binding groove formed by the docking domains of the RII dimer (Fig. 1*A*) (5, 6). Competitor peptides that mimic the AKAP side of this protein-protein interface universally uncouple PKA anchoring irrespective of which AKAP is involved (18). High-resolution crystal structures of RII-AKAP peptide complexes indicate that non-conserved side chains in the AKAP helix bond with Ile-3, Ile-5, Thr-10, and Gln-14 of each RIIα protomer (Fig. 1*B*). We reasoned that systematically engineering these positions might introduce AKAP-selective binding determinants into RII.

On the basis of this postulate, we devised a phage display selection for AKAP-specific RII variants. The RIIα fragment (residues 1–45) encompassing the D/D domain was fused to the C terminus of the T7 bacteriophage capsid protein via a 10-amino acid linker (supplemental Fig. S1*A*). In pilot studies, GST-AKAP79 recovered 300-fold more RII phage compared with GST alone (supplemental Fig. S1*B*). In a more stringent test, successive rounds of binding to an immobilized AKAP79 fragment followed by phage amplification selected native RII phage from a starting pool that contained a 1000-fold molar excess of an anchoring-defective RII form (T17W) (supplemental Fig. S1, *C–F*). Taken together, these experiments attest to the feasibility of this approach.

Next, RII D/D domain inserts were amplified from a DNA template with random nucleotides encoding positions 3, 5, 10, and 14. RII mutant sequences were packaged into phage to construct a T7 capsid display library with >99% of the 160,000 (4^20^) possible RII mutant sequences (25). High-throughput sequencing confirmed that every amino acid was represented at positions 3, 5, 10, and 14 in RII (Fig. 1*C*, *red squares*) and that the wild-type amino acids persisted in the remainder of the fragment (Fig. 1*C*, *blue triangles*). Further inspection of the high-throughput sequencing data confirmed that nucleotide variation was limited to the codons of interest (Fig. 1*D*), that the majority of RII phage encoded mutations at four positions compared with the wild type (Fig. 1*E*), and that there was comprehensive coverage of all of the possible RII mutant sequences (Fig. 1*F*).

##### Competitive Screening for AKAP-selective RII Mutants

**FIGURE 2.**
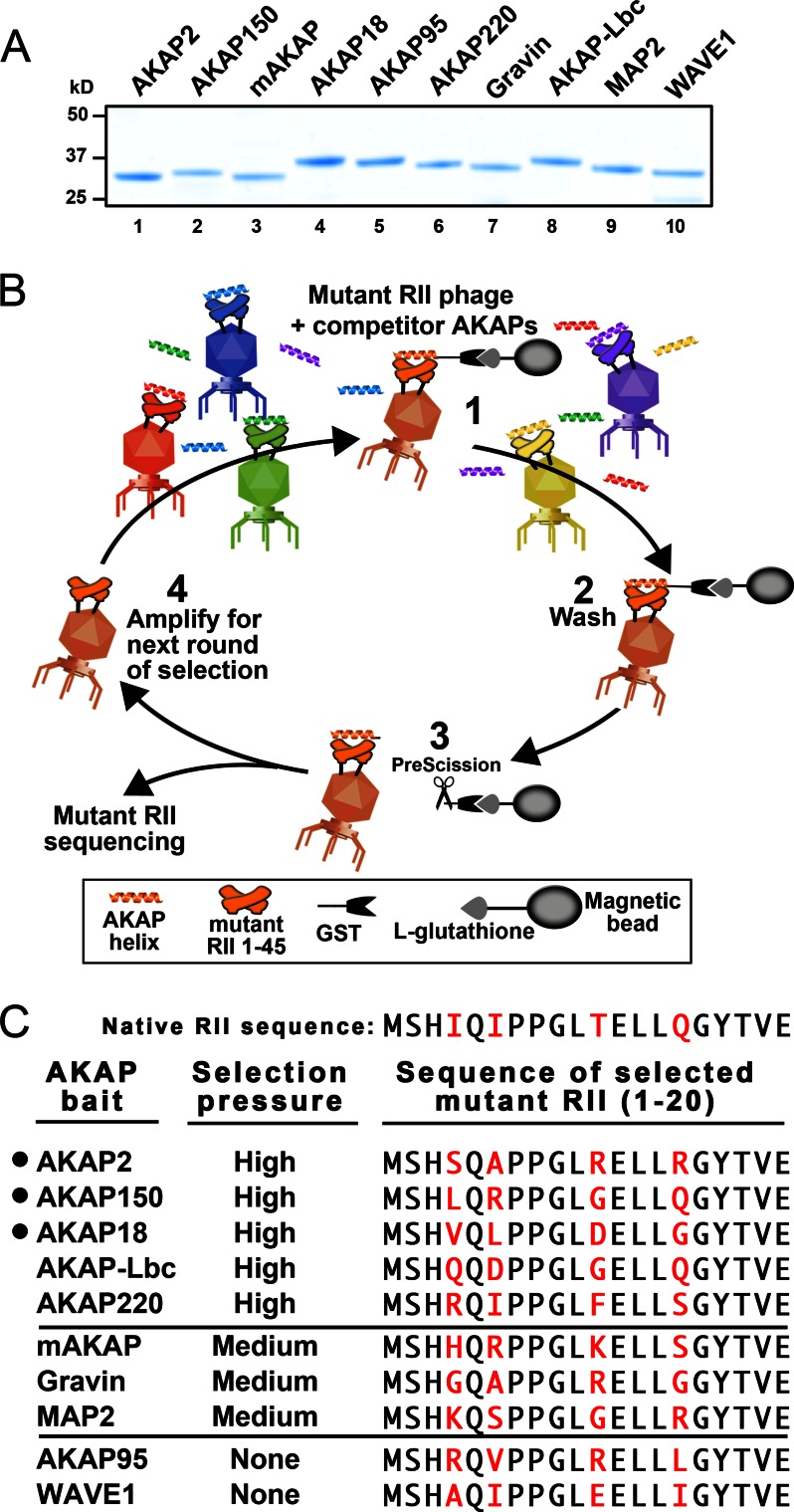
**Screening of AKAP-selective RII variants by phage selection.** *A*, Coomassie Blue-stained SDS-polyacrylamide gel of GST-AKAP anchoring helix fragments. *B*, four-step iterative scheme for enrichment of RII variant phage that are selective for a single AKAP. *C*, predominant RII variant sequences following phage selection with each of the 10 different AKAP baits. High-selection pressure was a 5000-fold molar excess of each competitor AKAP helix, medium-selection pressure was a 250-fold molar excess of the competitors, and *None* was the absence of competitor fragments.

Isolation of phage expressing AKAP-selective RII variants involved competitive screening using a panel of purified AKAP fragments (Fig. 2*A* and supplemental Table S1). This procedure was conducted in four steps. First, glutathione magnetic beads were charged with the GST-AKAP fragment of interest. This immobilized bait was incubated with the phage library in the presence of free competitor anchoring helices from the 15 AKAPs (Fig. 2*B*, *step 1*). A wash phase removed free and competitor AKAP-associated phage (Fig. 2*B*, *step 2*). PreScission protease cleavage liberated RII-AKAP phage complexes from the glutathione beads (Fig. 2*B*, *step 3*). Recovered phage were amplified in step 4 to serve as the input for the next round of selection. This stepwise cycle was repeated six to eight times until the selected material converged into a single RII sequence as determined by Sanger sequencing (supplemental Table S2).

This screening process was performed with 10 different GST-AKAP fragments as bait. Predominant RII variant sequences emerged for AKAP2, AKAP150, AKAP18, AKAP-Lbc, and AKAP220 when screens were performed under high-selection pressure (5000-fold molar excess of each competitor AKAP) (Fig. 2*C*, *upper*). Phage recovery was at background levels for the remaining five AKAPs. However, RII mutant sequences for mAKAP, Gravin, and MAP2 were forthcoming when screens were performed with a less stringent selection protocol (250-fold molar excess of each competitor AKAP) (Fig. 2*C*, *middle*). Isolation of RII variants for the low-affinity anchoring proteins AKAP95 and WAVE1 were successful in the absence of competitor AKAP peptides (Fig. 2*C*). Collectively, our screening process yielded 10 unique RII variant sequences selective for individual AKAPs (Fig. 2*C*, *right*). Each modified sequence contains substitutions at position 3, 5, 10, or 14 of RII (Fig. 2*C*, *right*).

##### R_Select_ Subunits Exhibit AKAP Selectivity

**FIGURE 3.**
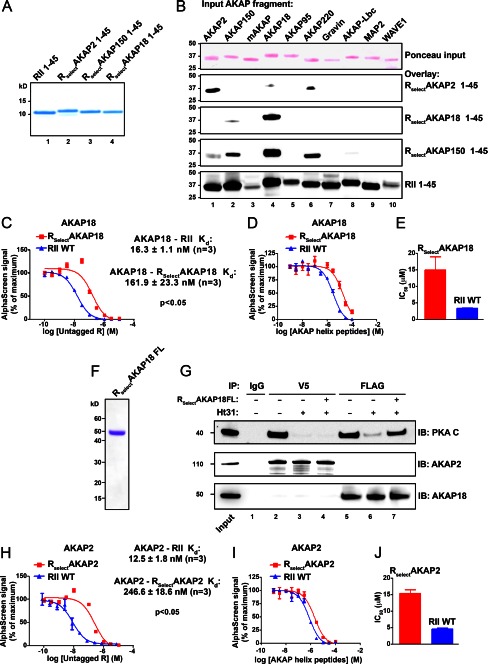
**R_Select_ subunits exhibit AKAP selectivity *in vitro*.** *A*, Coomassie Blue-stained SDS-polyacrylamide gel of purified R_Select_ fragments (residues 1–45). *B*, RII overlay of a panel of 10 GST-AKAP fusion proteins (Ponceau-stained; *first panel*) using the D/D domain fragments shown in *A*: R_Select_AKAP2(1–45) (*second panel*), R_Select_AKAP18(1–45) (*third panel*), R_Select_AKAP150(1–45) (*fourth panel*), and wild-type RII(1–45) (*fifth panel*). *C*, AlphaScreen competition binding assay for AKAP18 with D/D domain fragments of either R_Select_AKAP18 (*red squares*) or WT RII (*blue triangles*) and increasing concentrations of the appropriate untagged R subunit. The *K_d_* (*n* = 3) for AKAP18 interaction with either R_Select_AKAP18 (*red squares*) or WT RII (*blue triangles*) is shown. *D*, competition binding assay for AKAP18 with D/D domain fragments of either R_Select_AKAP18 (*red squares*) or WT RII (*blue triangles*) in the presence of increasing concentrations of a competitor AKAP peptide mixture. *E*, the IC_50_ (*n* = 3) of the competitor AKAP peptide mixture for AKAP18 associated with either R_Select_AKAP18 (*red bar*) or WT RII (*blue bar*). *F*, Coomassie Blue-stained SDS-polyacrylamide gel of the purified full-length RII mutant (R_Select_AKAP18FL). *G*, immunoprecipitations (*IP*) with HEK293 cell lysates were performed in the presence (*lanes 4* and *7*) and absence (*lanes 1–3*, *5*, and *6*) of R_Select_AKAP18FL using IgG (*lane 1*), anti-V5 antibody (*lanes 2–4*; for V5-AKAP2), or anti-FLAG antibody (*lanes 5–7*; for FLAG-AKAP18δ). These were done either with Ht31 (*lanes 3*, *4*, *6*, and *7*) or without Ht31 (*lanes 1*, *2*, and *5*). Anchoring and coprecipitation of the modified PKA holoenzyme were tested by immunoblotting (*IB*) for the PKA C subunit. Immunoprecipitation of AKAP18δ (anti-FLAG antibody), AKAP2 (anti-V5 antibody), and the PKA C subunit was confirmed by immunoblotting (*middle* and *lower panels*). *H*, AlphaScreen competition binding assay for AKAP2 with either R_Select_AKAP2 (*red squares*) or WT RII (*blue triangles*) and increasing concentrations of the appropriate untagged R subunit. The *K_d_* (*n* = 3) for AKAP2 interaction with either R_Select_AKAP2 or WT RII is indicted. *I*, AlphaScreen competition binding assay for AKAP2 with either R_Select_AKAP2 (*red squares*) or WT RII (*blue triangles*) in the presence of increasing concentrations of a competitor AKAP peptide mixture. *J*, the IC_50_ (*n* = 3) of the competitor AKAP peptide mixture for AKAP2 associated with either R_Select_AKAP2 (*red bar*) or WT RII (*blue bar*).

Biochemical validation of AKAP selectivity was limited to engineered RII(1–45) variants with a preference for AKAP2, AKAP150, and AKAP18. These proteins were designated R_Select_ (for RII-AKAP-Selective) subunits. Each C-terminally V5-tagged R_Select_ D/D domain was expressed in bacteria and purified by nickel affinity and gel filtration chromatography (Fig. 3*A*). The fidelity of these R_Select_ D/D domain fragments was tested by far-Western blotting against a collection of the PKA-anchoring domains from 10 different AKAPs (Fig. 3*B*, *first panel*). R_Select_AKAP2 and R_Select_AKAP18 preferentially bound to their cognate AKAP-binding partners (Fig. 3*B*, *second* and *third panels*). In contrast, R_Select_AKAP150 exhibited an intermediate selectivity with a binding preference for AKAP150, AKAP18, and AKAP220 (Fig. 3*B*, *fourth panel*). Control experiments confirmed that wild-type RII(1–45) bound each AKAP fragment in the overlay assay (Fig. 3*B*, *fifth panel*).

We further investigated the binding affinities of R_Select_AKAP2 and R_Select_AKAP18 using AlphaScreen. Dissociation constants were measured for AKAP18 using either RII or R_Select_AKAP18 as the ligand. Wild-type RII bound AKAP18 with a *K_d_* of 16.3 ± 1.1 nm (*n* = 3), whereas R_Select_AKAP18 exhibited a lower affinity for the anchoring protein (*K_d_* 161.9 ± 23.3 nm (*n* = 3), *p* < 0.05) (Fig. 3*C*). However, in the presence of a mixture of competitor AKAP peptides, the AKAP18-R_Select_AKAP18 complex was 4.4-fold more resistant to disruption than the wild-type complex (*n* = 3, *p* = 0.043) (Fig. 3, *D* and *E*). Thus, although R_Select_AKAP18 binds its anchoring protein with reduced affinity, this engineered form has greater selectivity for AKAP18 compared with wild-type RII.

We took advantage of this latter property to further test the efficacy of binding of R_Select_AKAP18 to its preferred anchoring protein. We reasoned that PKA holoenzymes formed with R_Select_AKAP18 should remain attached to the anchoring protein, even in the presence of the PKA-anchoring disruptor peptide Ht31 (24). Mutations were introduced at positions 3, 5, 10, and 14 in the full-length R subunit to generate R_Select_AKAP18FL (Fig. 3*F*). This modified PKA holoenzyme remained anchored to AKAP18 in the presence of Ht31 (Fig. 3*G*). Western blotting detected the C subunit in AKAP18 immune complexes (Fig. 3*G*, *upper panel*, *lane 7*). Control experiments confirmed that R_Select_AKAP18FL did not enhance the co-fractionation of the C subunit with AKAP2 under the same conditions (Fig. 3*G*, *lanes 2–4*). Likewise, AlphaScreen measurements revealed reduced binding affinity but enhanced selectivity of R_Select_AKAP2 for its preferred anchoring protein compared with wild-type RII (Fig. 3, *H–J*). Wild-type RII subunits (*K_d_* = 12.5 ± 1.8 nm) (Fig. 3*H*, *blue triangles*) bound AKAP2 with significantly (*p* < 0.05) higher affinity than R_Select_AKAP2 (*K_d_* = 246.6 ± 18.6 nm) (Fig. 3*H*, *red squares*). However, whereas a competitor AKAP peptide mixture disrupted the interaction between AKAP2 and wild-type RII with an IC_50_ = 0.68 ± 0.05 μm (Fig. 3, *I* and *J*, *blue*), the AKAP2-R_Select_AKAP2 complex was significantly more resistant to disruption by 3.4-fold (*n* = 3, *p* < 0.05) according to the IC_50_ (2.3 ± 0.16 μm) (Fig. 3, *I* and *J*, *red*).

**FIGURE 4.**
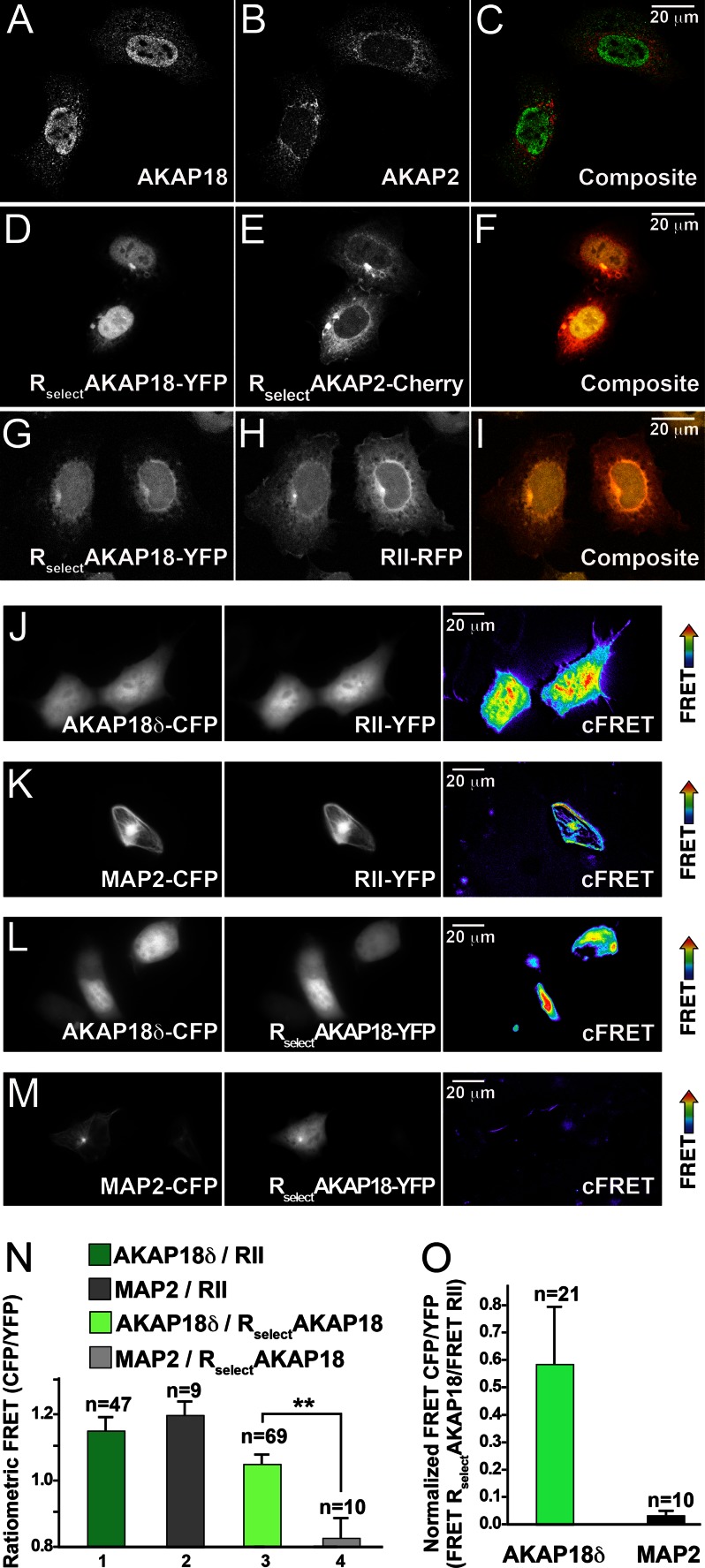
**R_Select_ subunits exhibit AKAP selectivity in cells.** *A–I*, confocal images of full-length AKAPs and R_Select_ D/D domain fragments show their subcellular location. Immunofluorescent images show HEK293 cells cotransfected with FLAG-AKAP18δ (*A*; *green* in *C*) and AKAP2-V5 (*B*; *red* in *C*). Fluorescent images show R_Select_AKAP18-YFP (*D*; *yellow* in *F*) and R_Select_AKAP2-Cherry (*E*; *red* in *F*), which were coexpressed with AKAP18δ and AKAP2. Fluorescent images show R_Select_AKAP18-YFP (*G*; *yellow* in *I*) and RII-RFP (*H*; *red* in *I*), which were coexpressed with AKAP18δ. *J–M*, CFP-YFP FRET imaging of the following RII-AKAP or R_Select_AKAP18 pairs in HEK293 cells: AKAP18δ-CFP and RII-YFP (*J*), MAP2-CFP and RII-YFP (*K*), AKAP18δ-CFP and R_Select_AKAP18-YFP (*L*), and MAP2-CFP and R_Select_AKAP18-YFP (*M*). Images were acquired for donor CFP (*left panels*) and acceptor YFP (*middle columns*), and corrected FRET images are presented (*right panels*). *N*, ratiometric quantification of intermolecular FRET pairs as described for *J–M*. **, *p* < 0.01. *O*, quantification of FRET signals in AKAP18δ-CFP/R_Select_AKAP18-YFP and MAP2-CFP/R_Select_AKAP18-YFP pairs normalized to AKAP18δ-CFP/RII-YFP and MAP2-CFP/RII-YFP, respectively.

Most AKAPs contain targeting motifs that associate with structural proteins or cellular membranes (26). We exploited this property to validate the cellular efficacy of R_Select_AKAP2 and R_Select_AKAP18 (Fig. 4). Immunofluorescence staining of fixed HEK293 cells showed AKAP2 enriched in perinuclear and reticular regions (17), whereas AKAP18δ is concentrated in the nucleus (27) (Fig. 4, *A* and *B*). The segregation of these AKAP signals can be seen in the composite image (Fig. 4*C*). These staining patterns were recapitulated in cells cotransfected with genetically encoded fluorescent R_Select_ D/D domain fragments (Fig. 4, *D–F*): R_Select_AKAP2-Cherry adopted a reticular staining pattern (Fig. 4*E*), whereas R_Select_AKAP18-YFP was predominantly nuclear (Fig. 4*D*). The nuclear localization of the R_Select_AKAP18 D/D domain was retained in the presence of wild-type RII-RFP (Fig. 4, *G–I*). The important conclusion that can be drawn from these studies is that these R_Select_ fragments have identical subcellular distributions to their AKAP-binding partners, implying that they can find their cognate AKAPs *in situ*.

To further test the selectivity of R_Select_AKAP18, we employed FRET to evaluate its binding to AKAP18δ or the PKA-anchoring protein MAP2 (28). This approach allows detection of protein proximity below the diffraction limit of light in living cells (29). As expected, both AKAP18δ-CFP and MAP2-CFP were capable of eliciting a FRET signal when paired with wild-type RII-YFP (Fig. 4, *J*, *K*, and *N*, *bars 1* and *2*). However, only AKAP18δ-CFP was capable of FRET when paired with R_Select_AKAP18-YFP (Fig. 4, *L*, *M*, and *N*, *bars 3* and *4*). Comparison of R_Select_AKAP18 FRET/RII FRET ratios confirmed that R_Select_AKAP18 preferentially recognizes AKAP18δ (0.57 ± 0.22) over MAP2 (0.03 ± 0.02) (Fig. 4*O*). Taken together, the far-Western blotting and fixed- and live-cell imaging data validate the selectivity of these R_Select_ molecules.

##### Molecular Basis for High-affinity RII Interaction with AKAP18

**FIGURE 5.**
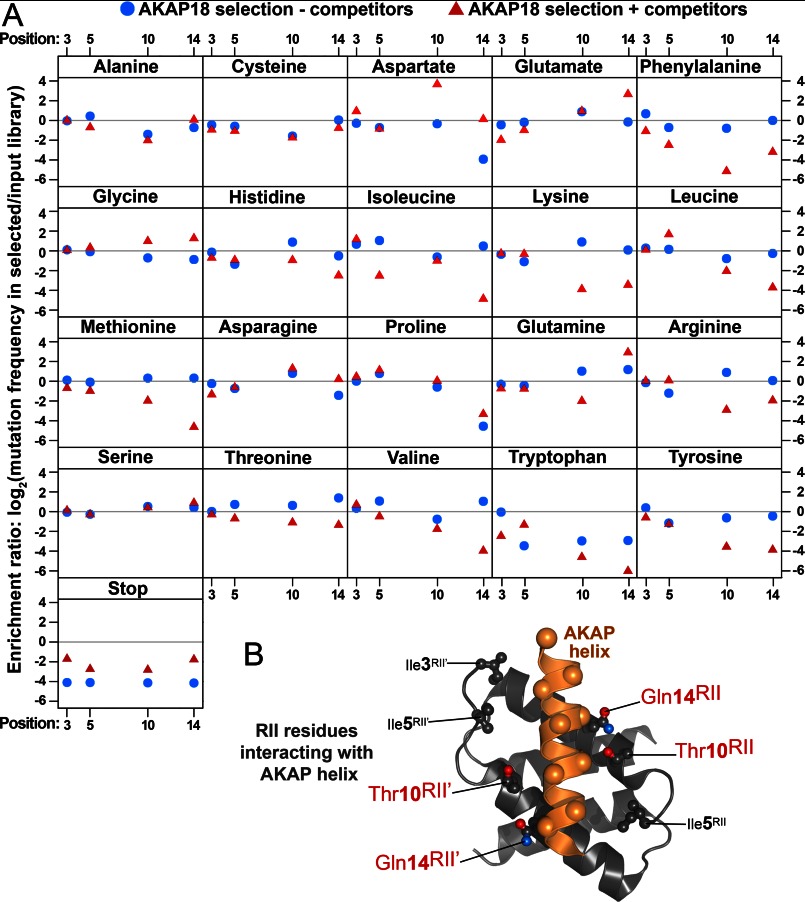
**Sequence-function plot of RII variant selection with AKAP18.** *A*, the relative changes in the frequency of each amino acid at the four variable RII positions are shown after three rounds of selection using GST-AKAP18 in either the presence (*red triangles*) or absence (*blue circles*) of competitor AKAP peptides. Amino acid mutations that increased in frequency relative to the input library have positive log-transformed enrichment ratios as a consequence of improved binding. *B*, structural representation of positions 3, 5, 10, and 14 in wild-type RII in relation to an AKAP anchoring helix (*orange*).

Systematic amino acid substitution at all positions of 16-residue AKAP helices has been invaluable in identifying determinants for R subunit interaction by immobilized peptide array (5, 18, 30). This experimental strategy was not feasible for analysis of the reciprocal binding surface because correct folding of the RII D/D domain is necessary to create the four-helix AKAP-binding groove. Therefore, high-throughput DNA sequencing of RII variant libraries before and after selection using AKAP18 (23) was utilized for a comprehensive structure-function analysis of the RII binding surface (Fig. 5*A*, *blue circles*). We reasoned that phage selection with AKAP18 alone could reveal which amino acids on RII favor high-affinity interaction with this anchoring protein. After three rounds of selection using AKAP18 as bait, sequence-function maps were compiled (Fig. 5*A*). This analysis indicated a change in frequency of amino acids at positions 3, 5, 10, and 14 in RII upon successive rounds of selection. Although the wild-type residue Ile was present at position 3, other side chains were also tolerated. The branched aliphatic amino acids Ile and Val were enriched at position 5. These data confirm that positions 3 and 5 in RII contribute to the integrity of the hydrophobic binding surface of the D/D domain (5, 6, 30). In contrast, side chains with the capacity to hydrogen bond emerged at positions 10 and 14 (Fig. 5*A*, *blue circles*). Hence, we conclude that these hydrophilic interactions enhance the recruitment of AKAP18 (Fig. 5*B*).

##### Molecular Basis for R_Select_ Binding Preference

**FIGURE 6.**
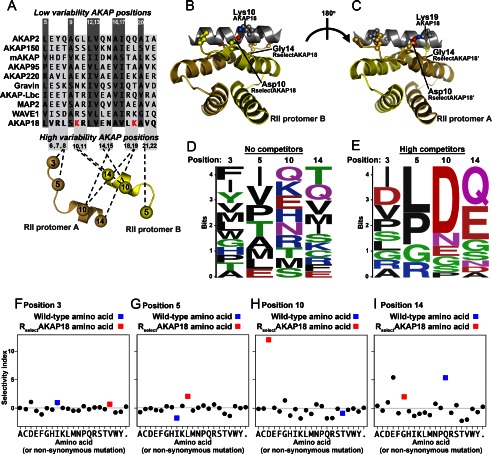
**Structural basis of R_Select_ selectivity** *A*, alignment of AKAP anchoring helices. Variable positions are shown in *light gray*, and highly conserved small aliphatic residues are shown in *dark gray* (*upper*). RII protomer residues 3, 5, 10, and 14 are shown below with *dashed lines* indicating interaction with variable AKAP positions. *B* and *C*, structural representations of a structural model of R_Select_AKAP18 in complex with the anchoring helix of AKAP18. *D* and *E*, Logo plots of an early round of selection with AKAP18 in the absence (*D*) or presence (*E*) of competitor AKAP peptides. Amino acid mutations that increased in frequency after selection were used to generate a Logo plot, where the height of each amino acid indicates its frequency at that position (WebLogo). The selectivity index for each amino acid substitution is shown at positions 3 (*F*), 5 (*G*), 10 (*H*), and 14 (*I*) in the RII sequence. *Black circles* indicate selectivity index scores, *blue squares* denote wild-type RII amino acids, and *red squares* denote R_Select_AKAP18 amino acids.

Three-dimensional structures of RII-AKAP complexes (5, 6) provide a molecular framework for understanding the binding preferences of R_Select_ proteins. The likely contact points between residues 3, 5, 10, and 14 of RII and the reciprocal side chains on an AKAP helix (high-variability positions) are shown in Fig. 6*A*. A structural model of this protein-protein interface was assembled by substitution and refinement of the 1.3 Å RII dimer-AKAP-*is* crystal structure (Fig. 6, *B* and *C*) (5). One striking feature is that Lys-10 and Lys-19, which project from alternate sides of the AKAP18 amphipathic helix, are optimally aligned to form salt bridge interactions with Asp-10 in each R_Select_AKAP18 protomer (Fig. 6, *B* and *C*). Scrutiny of the aligned RII-binding sequences from 10 AKAPs (Fig. 6*A*) revealed that only AKAP18 incorporates lysines on both sides of its anchoring helix. This could explain why aspartate was selected at position 10 in R_Select_AKAP18. Interestingly, AKAP18 is unique in orienting additional leucines at positions 8 and 18 in its anchoring helix (Fig. 6*A*). Our structural model suggests that these aliphatic side chains interact with Val-3 and Leu-5 in R_Select_AKAP18 (Fig. 6, *B* and *C*). Molecular models were also compiled for R_Select_AKAP2 and R_Select_AKAP150 complexes (supplemental Fig. S2). Each simulation predicts that increased complementarity between variable R_Select_ positions and cognate AKAP side chains is an important component of discrimination that is introduced into these engineered RII subunits. For example, Arg-10 and Arg-14 on each R_Select_AKAP2 protomer are positioned to hydrogen bond with Gln-14, Asn-15, Gln-18, and Gln-19 in AKAP2 (supplemental Fig. S2, *A–C*). Likewise, Gln-10 in R_Select_AKAP150 can form a hydrogen-bonding network with Ser-10 and Lys-14 in AKAP150 (supplemental Fig. S2, *A* , *D* , and *E*). This latter model also predicts that Arg-5 in R_Select_AKAP150 forms a salt bridge with Glu-18 in AKAP150 (supplemental Fig. S2, *A*, *D*, and *E*).

To consolidate predictions from the AKAP18-R_Select_AKAP18 structural model in an empirical manner, we performed further sequence-function analysis in the presence of competitor AKAP peptides. The enrichment ratios for each amino acid at positions 3, 5, 10, and 14 in RII in an early round of selection in the presence of competitors are shown in Fig. 5*A* (*red triangles*). Inclusion of competitor peptides increased the stringency of selection. This enhanced the selection of leucine at position 5, aspartate at position 10, and glutamate at position 14 in RII (Fig. 6, *D* and *E*). Selectivity plots were calculated by dividing AKAP18 enrichment ratios in the presence of competitors by the enrichment ratios in the absence of competitors (Fig. 6, *F–I*). These plots show that Leu-5 and Asp-10 are the most AKAP18-discerning side chains (Fig. 6, *G* and *H*). Conversely, these residues are not strongly selected in the absence of competitors (Fig. 5*A*, *blue circles*, and Fig. 6*D*). This implies that the inclusion of Leu-5 and Asp-10 has a negative effect on R_Select_AKAP18 binding to other AKAP family members. This conclusion is consistent with the results of the R subunits overlays (Fig. 3*B*) and *K_d_* measurements (Fig. 3, *E* and *F*), which showed that R_Select_AKAP18 bound to AKAP18 with lower affinity than wild-type RII in the absence of other AKAPs. Although aspartate is the most AKAP18-selective residue at position 14 in this analysis (Fig. 6*I*), the predominant R_Select_AKAP18 sequence that emerged after eight rounds of selection contains glycine at position 14. This suggests that one (but not two) negative charges on alternate sides of the AKAP18 anchoring helix provide optimal selectivity and that Gly-14 may cooperate with Asp-10 to enable formation of salt bridges between the acidic residue and lysines on either side of the AKAP18 anchoring helix (Fig. 6, *B* and *C*). In sum, the modeling and high-throughput sequencing analyses support our initial hypothesis that mutations at RII positions 3, 5, 10, and 14 can impart AKAP-selective properties.

## DISCUSSION

On the basis of high-resolution structures of the AKAP-PKA interface, we generated a phage library bearing 160,000 variants of the RII D/D domain. Following the protocol outlined in Fig. 2*B*, we enriched for RII variants with selectivity for a particular AKAP. The efficacy of this approach is demonstrated in Figs. 3 and 4 by the preferential association of R_Select_ subunits with their cognate AKAPs *in vitro* and *in vivo*. When combined with structural modeling, high-throughput sequencing analysis has provided a clearer understanding of which side chains on RII enhance association with individual AKAPs. Comparative analysis of AKAP18 selections in the presence and absence of competitors showed that residues in the optimal R_Select_ sequence are both compatible with residues at the high-variability positions in the target AKAP and incompatible with resides at the equivalent positions in off-target AKAPs.

In addition, the sequence-function analysis presented in Fig. 5 revealed that hydrophilic contacts facilitated by positions 10 and 14 in RII are more critical for interaction with AKAP18 compared with positions 3 and 5. Inspection of crystal structures of the RII-AKAP-*is* and RII-D-AKAP2 complexes (5, 6) revealed that Thr-10 on a single RII protomer forms a water-mediated hydrogen bond with the backbone carbonyl of Ile-12 in the AKAP helix. Thr at position 10 favors high-affinity interaction with AKAP18 in comparison with the sterically similar side chains Val and Ile (Fig. 5). Therefore, this hydrophilic contact is likely to be an important component of all RII-AKAP interfaces. The influence of this hydrophilic quadrad that comprises the core of the D/D domain should be factored into attempts to generate small molecules that disrupt RII-AKAP interactions (31).

Our intermolecular FRET measurements indicate that R_Select_ subunits are viable chemical biology reagents that selectively associate with AKAPs in living cells. R_Select_AKAP18-YFP and AKAP18δ-CFP must be within ∼80 Å to permit FRET (Fig. 4, *J–O*) (29). Because C-terminal epitope tagging does not impair the ability of R_Select_AKAP18 to recognize its anchoring protein inside cells, chemical or biological reporters may be fused to R_Select_ subunits. Fusion of chemical moieties such as photoactivatable GFP or phosphorylation-sensitive FRET reporters (32) may ultimately permit the monitoring of movement and the activity of subpopulations of PKA associated with a given AKAP. Similarly, fusion of biologically active molecules such as the PKA C subunit or cAMP phosphodiesterase to a given R_Select_ subunit may enable controlled up- or down-regulation of PKA activity within the immediate vicinity of a particular AKAP complex. Prospective delivery methods include transfection, genomic incorporation (33), and inclusion of purified R_Select_ D/D domain in the patch pipette in a manner analogous to generic PKA-anchoring disruptor reagents (5). Because wild-type RII binds to AKAP2 and AKAP18 with significantly higher affinity than the respective R_Select_ subunits for these AKAPs, the best approach may be to apply the R_Select_ subunits in tandem with Ht31 (Fig. 3*H*) to determine whether individual AKAPs are sufficient for targeting PKA to particular ion channels. Future biological studies to address using R_Select_-based reagents include establishing the relative contributions of individual AKAP-anchored subpopulations of PKA that regulate synaptic transmission (34, 35) and control cardiac excitation-contraction coupling (36). AKAP-selective PKA disruptors have also been conjectured as forerunners of the next generation of drugs that target the β-adrenergic system in the heart (37). Hence, the continued development of R_Select_ reagents may contribute to target validation of these new therapeutic agents.

In a broader context, we anticipate that our structure-based phage selection procedure may enable development of synthetic biology reagents for studying other functionally important signaling protein interactions. The most promising candidate interfaces are those for which high-resolution structural information is available to guide the design of mutant libraries. Theoretically, the T7Select system could also accommodate more complex phage libraries containing at least five randomized positions (20^5^ = 3.2 × 10^6^ combinations) within the target sequence. Display of molecules larger than 45 amino acids is also possible using T7 bacteriophage. Thus, it may be possible to design mutant libraries for intact proteins such as phosphatase C subunits that selectively recognize protein phosphatase 1-targeting subunits by virtue of the RV*x*F motif (38) or calcineurin/protein phosphatase 2B-anchoring proteins that work through PI*X*IT motifs (11, 12). The development of such reagents will facilitate spatial manipulation of enzyme activities and aid in our understanding of normal and pathological signaling processes.

## Footnotes

3 The abbreviations used are:

AKAP: A-kinase anchoring protein
D/D: docking and dimerization
RII: regulatory type II.

## References

[1] Pawson C. T. Scott J. D. (2010) Signal integration through blending, bolstering and bifurcating of intracellular information. Nat. Struct. Mol. Biol. 17, 653–658 20495562 10.1038/nsmb.1843 PMC3086636

[2] Welch E. J. Jones B. W. Scott J. D. (2010) Networking with AKAPs: context-dependent regulation of anchored enzymes. Mol. Interv. 10, 86–97 20368369 10.1124/mi.10.2.6 PMC2895371

[3] Roy J. Cyert M. S. (2009) Cracking the phosphatase code: docking interactions determine substrate specificity. Sci. Signal. 2, re9 19996458 10.1126/scisignal.2100re9

[4] Gold M. G. Barford D. Komander D. (2006) Lining the pockets of kinases and phosphatases. Curr. Opin. Struct. Biol. 16, 693–701 17084073 10.1016/j.sbi.2006.10.006

[5] Gold M. G. Lygren B. Dokurno P. Hoshi N. McConnachie G. Taskén K. Carlson C. R. Scott J. D. Barford D. (2006) Molecular basis of AKAP specificity for PKA regulatory subunits. Mol. Cell 24, 383–395 17081989 10.1016/j.molcel.2006.09.006

[6] Kinderman F. S. Kim C. von Daake S. Ma Y. Pham B. Q. Spraggon G. Xuong N. H. Jennings P. A. Taylor S. S. (2006) A dynamic mechanism for AKAP binding to RII isoforms of cAMP-dependent protein kinase. Mol. Cell 24, 397–408 17081990 10.1016/j.molcel.2006.09.015 PMC1855097

[7] Bauman A. L. Soughayer J. Nguyen B. T. Willoughby D. Carnegie G. K. Wong W. Hoshi N. Langeberg L. K. Cooper D. M. Dessauer C. W. Scott J. D. (2006) Dynamic regulation of cAMP synthesis through anchored PKA-adenylyl cyclase V/VI complexes. Mol. Cell 23, 925–931 16973443 10.1016/j.molcel.2006.07.025 PMC3941446

[8] Keely S. L. (1977) Activation of cAMP-dependent protein kinase without a corresponding increase in phosphorylase activity. Res. Commun. Chem. Pathol. Pharmacol. 18, 283–290 199922

[9] Egloff M. P. Johnson D. F. Moorhead G. Cohen P. T. Cohen P. Barford D. (1997) Structural basis for the recognition of regulatory subunits by the catalytic subunit of protein phosphatase 1. EMBO J. 16, 1876–1887 9155014 10.1093/emboj/16.8.1876 PMC1169791

[10] Terrak M. Kerff F. Langsetmo K. Tao T. Dominguez R. (2004) Structural basis of protein phosphatase 1 regulation. Nature 429, 780–784 15164081 10.1038/nature02582

[11] Gold M. G. Stengel F. Nygren P. J. Weisbrod C. R. Bruce J. E. Robinson C. V. Barford D. Scott J. D. (2011) Architecture and dynamics of an A-kinase anchoring protein 79 (AKAP79) signaling complex. Proc. Natl. Acad. Sci. U.S.A. 108, 6426–6431 21464287 10.1073/pnas.1014400108 PMC3081024

[12] Li H. Pink M. D. Murphy J. G. Stein A. Dell'Acqua M. L. Hogan P. G. (2012) Balanced interactions of calcineurin with AKAP79 regulate Ca^2+^-calcineurin-NFAT signaling. Nat. Struct. Mol. Biol. 19, 337–345 22343722 10.1038/nsmb.2238 PMC3294036

[13] Bashor C. J. Helman N. C. Yan S. Lim W. A. (2008) Using engineered scaffold interactions to reshape MAP kinase pathway signaling dynamics. Science 319, 1539–1543 18339942 10.1126/science.1151153

[14] Wu Y. I. Frey D. Lungu O. I. Jaehrig A. Schlichting I. Kuhlman B. Hahn K. M. (2009) A genetically encoded photoactivatable Rac controls the motility of living cells. Nature 461, 104–108 19693014 10.1038/nature08241 PMC2766670

[15] Rosenmund C. Carr D. W. Bergeson S. E. Nilaver G. Scott J. D. Westbrook G. L. (1994) Anchoring of protein kinase A is required for modulation of AMPA/kainate receptors on hippocampal neurons. Nature 368, 853–856 8159245 10.1038/368853a0

[16] Lester L. B. Langeberg L. K. Scott J. D. (1997) Anchoring of protein kinase A facilitates hormone-mediated insulin secretion. Proc. Natl. Acad. Sci. U.S.A. 94, 14942–14947 9405718 10.1073/pnas.94.26.14942 PMC25142

[17] Gold M. G. Reichow S. L. O'Neill S. E. Weisbrod C. R. Langeberg L. K. Bruce J. E. Gonen T. Scott J. D. (2012) AKAP2 anchors PKA with aquaporin-0 to support ocular lens transparency. EMBO Mol. Med. 4, 15–26 22095752 10.1002/emmm.201100184 PMC3272850

[18] Alto N. M. Soderling S. H. Hoshi N. Langeberg L. K. Fayos R. Jennings P. A. Scott J. D. (2003) Bioinformatic design of A-kinase anchoring protein *in silico*: a potent and selective peptide antagonist of type II protein kinase A anchoring. Proc. Natl. Acad. Sci. U.S.A. 100, 4445–4450 12672969 10.1073/pnas.0330734100 PMC153575

[19] Carlson C. R. Lygren B. Berge T. Hoshi N. Wong W. Taskén K. Scott J. D. (2006) Delineation of type I protein kinase A-selective signaling events using an RI anchoring disruptor. J. Biol. Chem. 281, 21535–21545 16728392 10.1074/jbc.M603223200

[20] Grote A. Hiller K. Scheer M. Münch R. Nörtemann B. Hempel D. C. Jahn D. (2005) JCat: a novel tool to adapt codon usage of a target gene to its potential expression host. Nucleic Acids Res. 33, W526–W531 15980527 10.1093/nar/gki376 PMC1160137

[21] Emsley P. Lohkamp B. Scott W. G. Cowtan K. (2010) Features and development of Coot. Acta Crystallogr. D Biol. Crystallogr. 66, 486–501 20383002 10.1107/S0907444910007493 PMC2852313

[22] Fowler D. M. Araya C. L. Gerard W. Fields S. (2011) Enrich: software for analysis of protein function by enrichment and depletion of variants. Bioinformatics 27, 3430–3431 22006916 10.1093/bioinformatics/btr577 PMC3232369

[23] Fowler D. M. Araya C. L. Fleishman S. J. Kellogg E. H. Stephany J. J. Baker D. Fields S. (2010) High-resolution mapping of protein sequence-function relationships. Nat. Methods 7, 741–746 20711194 10.1038/nmeth.1492 PMC2938879

[24] Carr D. W. Stofko-Hahn R. E. Fraser I. D. Bishop S. M. Acott T. S. Brennan R. G. Scott J. D. (1991) Interaction of the regulatory subunit (RII) of cAMP-dependent protein kinase with RII-anchoring proteins occurs through an amphipathic helix binding motif. J. Biol. Chem. 266, 14188–14192 1860836

[25] Knight R. Yarus M. (2003) Analyzing partially randomized nucleic acid pools: straight dope on doping. Nucleic Acids Res. 31, e30 12626729 10.1093/nar/gng030 PMC152884

[26] Scott J. D. Pawson T. (2009) Cell signaling in space and time: where proteins come together and when they're apart. Science 326, 1220–1224 19965465 10.1126/science.1175668 PMC3041271

[27] Brown R. L. August S. L. Williams C. J. Moss S. B. (2003) AKAP7γ is a nuclear RI-binding AKAP. Biochem. Biophys. Res. Commun. 306, 394–401 12804576 10.1016/s0006-291x(03)00982-3

[28] Theurkauf W. E. Vallee R. B. (1982) Molecular characterization of the cAMP-dependent protein kinase bound to microtubule-associated protein 2. J. Biol. Chem. 257, 3284–3290 6277931

[29] Giepmans B. N. G. Adams S. R. Ellisman M. H. Tsien R. Y. (2006) The fluorescent toolbox for assessing protein location and function. Science 312, 217–224 16614209 10.1126/science.1124618

[30] Burns-Hamuro L. L. Ma Y. Kammerer S. Reineke U. Self C. Cook C. Olson G. L. Cantor C. R. Braun A. Taylor S. S. (2003) Designing isoform-specific peptide disruptors of protein kinase A localization. Proc. Natl. Acad. Sci. U. S. A. 100, 4072–4077 12646696 10.1073/pnas.2628038100 PMC153050

[31] Christian F. Szaszák M. Friedl S. Drewianka S. Lorenz D. Goncalves A. Furkert J. Vargas C. Schmieder P. Götz F. Zühlke K. Moutty M. Göttert H. Joshi M. Reif B. Haase H. Morano I. Grossmann S. Klukovits A. Verli J. Gáspár R. Noack C. Bergmann M. Kass R. Hampel K. Kashin D. Genieser H. G. Herberg F. W. Willoughby D. Cooper D. M. Baillie G. S. Houslay M. D. von Kries J. P. Zimmermann B. Rosenthal W. Klussmann E. (2011) Small molecule AKAP-protein kinase A (PKA) interaction disruptors that activate PKA interfere with compartmentalized cAMP signaling in cardiac myocytes. J. Biol. Chem. 286, 9079–9096 21177871 10.1074/jbc.M110.160614 PMC3058960

[32] Dodge-Kafka K. L. Soughayer J. Pare G. C. Carlisle Michel J. J. Langeberg L. K. Kapiloff M. S. Scott J. D. (2005) The protein kinase A anchoring protein mAKAP coordinates two integrated cAMP effector pathways. Nature 437, 574–578 16177794 10.1038/nature03966 PMC1636584

[33] Harvey C. D. Ehrhardt A. G. Cellurale C. Zhong H. Yasuda R. Davis R. J. Svoboda K. (2008) A genetically encoded fluorescent sensor of ERK activity. Proc. Natl. Acad. Sci. U.S.A. 105, 19264–19269 19033456 10.1073/pnas.0804598105 PMC2614750

[34] Tunquist B. J. Hoshi N. Guire E. S. Zhang F. Mullendorff K. Langeberg L. K. Raber J. Scott J. D. (2008) Loss of AKAP150 perturbs distinct neuronal processes in mice. Proc. Natl. Acad. Sci. U.S.A. 105, 12557–12562 18711127 10.1073/pnas.0805922105 PMC2527950

[35] Zhong H. Sia G. M. Sato T. R. Gray N. W. Mao T. Khuchua Z. Huganir R. L. Svoboda K. (2009) Subcellular dynamics of type II PKA in neurons. Neuron 62, 363–374 19447092 10.1016/j.neuron.2009.03.013 PMC2702487

[36] Lygren B. Carlson C. R. Santamaria K. Lissandron V. McSorley T. Litzenberg J. Lorenz D. Wiesner B. Rosenthal W. Zaccolo M. Taskén K. Klussmann E. (2007) AKAP complex regulates Ca^2+^ re-uptake into heart sarcoplasmic reticulum. EMBO Rep. 8, 1061–1067 17901878 10.1038/sj.embor.7401081 PMC2247390

[37] Wells J. A. McClendon C. L. (2007) Reaching for high-hanging fruit in drug discovery at protein-protein interfaces. Nature 450, 1001–1009 18075579 10.1038/nature06526

[38] Bollen M. Peti W. Ragusa M. J. Beullens M. (2010) The extended PP1 toolkit: designed to create specificity. Trends Biochem. Sci. 35, 450–458 20399103 10.1016/j.tibs.2010.03.002 PMC3131691

